# Ethical evaluation of AI-supported mental health applications

**DOI:** 10.3389/fpsyt.2026.1870948

**Published:** 2026-08-28

**Authors:** Ece Deveci, Perihan Elif Ekmekci

**Affiliations:** 1 Dr. Abdurrahman Yurtaslan Ankara Oncology Training and Research Hospital, Ankara, Türkiye; 2 Department of History of Medicine and Ethics, TOBB ETU Faculty of Medicine, Ankara, Türkiye

**Keywords:** artificial intelligence, mental health, medical ethics, chatbots, digital health, health technologies, psychiatry

## Abstract

**Background:**

Psychiatric ethics is a uniquely significant domain within the broader field of medical ethics, addressing complex issues such as confidentiality, privacy, informed consent, involuntary admission and treatment, and the intricacies of the physician-patient relationship. As artificial intelligence (AI) becomes increasingly integrated into psychiatric practice, including electronic health records, brain imaging systems, social media platforms, mobile applications, telepsychiatry, the Internet of Things, and therapeutic chatbots, it improves efficiency by aiding in the detection of prodromal phases of illnesses, facilitating patient follow-up, and fostering personalized treatment strategies; however, it also risks deepening existing ethical dilemmas and introducing new ones. It is vital that the algorithms and AI systems underpinning these applications are designed with ethical principles at their core, prioritizing patient benefit and avoiding harm.

**Methodology:**

A three-step approach was applied: (1) a narrative/conceptual literature review across PubMed/MEDLINE, Scopus, and Web of Science; (2) ethical and clinical analysis of four widely used AI-supported mental health applications (Wysa, Youper, Amaha, Yuna) across 14 categories; and (3) semi-structured interviews with five practicing psychiatrists, analyzed using grounded theory. Findings were interpreted through a two-part ethical framework: one layer grounded in psychiatric ethics norms, the other in Beauchamp and Childress’s four biomedical principles.

**Results/discussion:**

Application analysis showed gaps in transparency, accountability, crisis detection, and clinical accuracy. Analysis of the interviews revealed four main themes: Ontological mismatch with psychiatry, clinical role displacement, digital empathy limitations, and emotional responses to AI. These findings were structured into a conceptual framework at the normative, practical, and perceptual levels.

**Conclusion/perspectives:**

Despite the growing presence of AI in psychiatric care, there is a significant lack of developed ethical frameworks specifically tailored to AI-supported mental health applications, highlighting the urgent need for a standardized ethical evaluation framework that prioritizes patient well-being and autonomy. Future research should expand clinician samples, include patient and developer perspectives, and undertake longitudinal studies to examine the long-term safety of AI systems in psychiatry.

## 1 Introduction

Psychiatry is a discipline that focuses on patterns of emotion, thought, and behavior. By its nature, psychiatry encounters ethical dilemmas that are often more complex and profound than those in other fields of medicine (1). The core principles of medical ethics—autonomy, beneficence, non-maleficence, and justice (2)—are manifested in complex and nuanced ways within psychiatry. Therefore, psychiatric ethics occupies a distinctive position, addressing the unique challenges and nuances of psychiatric practice beyond the general principles of medical ethics. Issues such as confidentiality, privacy, informed consent, involuntary hospitalization and treatment, and the physician–patient relationship are among the primary areas of debate in psychiatric ethics (3). While aiming to prioritize patient welfare, psychiatrists often risk simultaneously compromising patient autonomy (4).

In recent years, as in other domains, the use of artificial intelligence (AI) technologies in healthcare has become increasingly widespread, particularly in diagnosis, treatment, and patient monitoring. While AI in healthcare holds promise for improving health systems and public health, it also raises concerns related to patient privacy, trust, bias, stigmatization, and accountability (5). Because these technologies process highly sensitive data related to individuals’ mental health, more rigorous ethical scrutiny is required.

In psychiatric practice, AI is increasingly being applied through electronic health records, neuroimaging tools, social media platforms, mobile applications, telepsychiatry, and the Internet of Things (6, 7). For instance, chatbot-based applications such as Wysa, Woebot, and Sanvello support stress management and emotion and thought monitoring while functioning as therapeutic conversational agents. While these applications offer benefits, such as early detection of prodromal phases of illness, enhanced patient monitoring, personalized treatment, and increased patient engagement in therapy (6–8), they also have the potential to intensify existing ethical dilemmas in psychiatry and introduce new concerns. These applications store and process large amounts of personal data, including patients’ medical histories and emotional states. Therefore, the secure storage, protection, and appropriate use of such data are essential for maintaining confidentiality and privacy (9). Another key concern is ensuring that the algorithms underlying these applications are developed in accordance with ethical principles, particularly beneficence and non-maleficence. Respecting autonomy also requires that patients are adequately informed and provide explicit consent. When patients understand and actively agree to their treatment, this not only supports their participation but also helps build trust, whether care is delivered by a human or an AI system.

Recent reports of adolescents interacting with AI chatbots before committing suicide have highlighted the urgent need to evaluate these technologies from a psychiatric ethics perspective. These cases demonstrate the serious risks that such systems may pose to vulnerable users. A recent BBC report described cases in which families linked prolonged chatbot use to worsening distress and suicide among adolescents. In some cases, chatbots failed to recognize or appropriately respond to suicidal thoughts, raising concerns about safety and oversight (10).

Since research on AI-supported mental health applications remains limited and a clear ethical framework is lacking in this area (11), there is an urgent need for a common ethical standard to safeguard patient welfare, mitigate risks, and protect autonomy (12). Furthermore, no established normative ethical frameworks currently exist for AI-supported applications in other healthcare domains.

The present study evaluated AI-supported mental health applications from a bioethical perspective. We combined a normative-analytical approach with qualitative insights and adopted an interdisciplinary approach. The study was conducted in three steps. First, a literature review was conducted to explore current debates and identify the key bioethical principles relevant to the study. This process highlighted the core principles—autonomy, privacy, non-maleficence, beneficence, justice, trust, veracity, and fidelity—as the foundation for the analysis. In the second stage, several AI-supported mental health applications currently in use were selected and analyzed through the lens of these principles to highlight their strengths and potential ethical risks. Finally, semi-structured interviews were conducted with psychiatrists to explore how these ethical concerns are experienced and perceived in practice. The interview data were analyzed thematically and then compared with the findings from the literature review and application analysis. Given the complexity of psychiatric ethics, this study adopted a two-layered approach. Ethical challenges were examined not only through core biomedical principles but also in relation to established psychiatric ethical norms. This helped ensure both theoretical and practical relevance. Overall, these three stages supported the main aims of this study: to identify ethical issues in AI-supported mental health applications, propose normative solutions, and map the existing literature on AI in psychiatry. This study was conducted in accordance with the principles of the Declaration of Helsinki. Ethical approval was granted by the institutional ethics committee. Ethical approval was granted by the TOBB University of Economics and Technology Scientific Research Ethics Committee (Protocol No. BAEK-15; Decision No. 13; approval date: October 2, 2024)

### 1.1 The use of artificial intelligence in psychiatry

In recent years, AI technologies have increasingly contributed to healthcare systems by assisting with diagnosis and patient monitoring. Their impact on psychiatry is particularly significant with respect to the early diagnosis and intervention of common psychiatric disorders (13).

AI-supported virtual psychotherapy applications developed through chatbots have advanced rapidly, with new tools emerging regularly. Such AI-based systems provide support to patients with mood and anxiety disorders, children with behavioral difficulties, and individuals without formal psychiatric diagnoses who nonetheless experience mental health concerns (14). Woebot, Wysa, Yuna, and similar programs engage users through short text or voice messages, simulating interactions with virtual psychotherapists. These applications aim to help users recognize emotional patterns, enhance resilience, reduce anxiety, and address sleep problems (14).

#### 1.1.1 Benefits

AI-supported interventions can have an important role in the early detection of mental health difficulties, reaching high-risk groups such as veterans and individuals with substance use disorders, and providing alternatives for those hesitant to seek traditional therapy because of stigma (15). In some cases, patients may respond more positively to robotic or virtual agents than to human therapists and may experience less shame when sharing sensitive information (16). For patients unfamiliar with healthcare systems, these tools may also improve treatment adherence, thereby strengthening the trust between patients and healthcare providers.

Another key advantage is efficiency. AI can triage and provide rapid access to support for individuals without urgent needs, allowing psychiatrists to focus on more complex or high-risk cases (14). Accessibility is another major benefit; AI tools can extend mental health services to rural or underserved areas, as well as to patients with financial constraints who cannot afford regular therapy (14). However, accessibility depends on devices and internet services, which may remain challenging in some rural communities and raise issues of fairness in access to the benefits of emerging technologies.

AI applications also offer constant availability, flexibility, and nonjudgmental support, making them particularly valuable for individuals seeking immediate assistance. Beyond individual applications, high-quality evidence from systematic reviews and meta-analyses indicates that AI-based conversational agents and digital mental health interventions can significantly reduce symptoms of anxiety, depression, and psychological distress, while improving accessibility and continuity of care (17, 18). Moreover, recent literature has highlighted the transformative potential of language models in psychotherapy, particularly for enhancing personalized and scalable mental health support (19).

Considering the increasing patient burden in hospitals and limited resources, well-designed AI-supported psychiatric tools may play an important role in improving access to and the quality of healthcare services.

#### 1.1.2 Ethical challenges

Despite their advantages, AI technologies in psychiatry are not universally welcomed by clinicians, and some may choose not to use them because of ethical or clinical concerns (14).

#### 1.1.3 Privacy, autonomy, and informed consent

The collection, processing, and storage of sensitive patient data in AI systems raise concerns regarding privacy and confidentiality. Ambiguity surrounding whether the interacting “agent” is a physician further complicates the adequacy of informed consent. Nevertheless, patients should be transparently informed about the existence and extent of AI’s role in their treatment, and explicit consent must be obtained to protect their autonomy. Transparency and clarity regarding AI participation may also enhance trust in healthcare providers and systems (20).

#### 1.1.4 Accountability

Sedlakova and Trachsel (21) argue that AI therapists cannot bear ethical responsibilities equivalent to those of human therapists because they lack autonomy and consciousness: AI cannot be considered as an equal partner in a conversation, as is the case with a human therapist. Instead, AI’s role should be restricted to specific functions. The absence of a clear accountability framework creates uncertainty regarding liability in cases of misdiagnosis or harmful outcomes (22).

#### 1.1.5 Non-maleficence and patient safety

The most serious concern with AI relates to the principle of non-maleficence: avoiding harm. While AI tools can provide support for mild conditions, they may fail in crises. There have already been tragic cases in which individuals engaged in AI chatbots before attempting or committing suicide (23). These examples reveal the dangers of systems that lack robust crisis intervention protocols. Without clear safeguards, technologies intended to provide comfort may instead cause harm.

#### 1.1.6 Data security

AI systems collect vast amounts of personal and sensitive health information, which increases the risk of data leakage (13). Fiske et al. (14) highlighted the risks of audio and video data leakage and emphasized the need for enhanced safeguards. Alfano et al. (20) highlight uncertainties surrounding data storage, including where, by whom, and for how long information is stored, as well as the risks of third-party sales following corporate acquisitions.

#### 1.1.7 Therapeutic relationship and empathy

Psychiatric care involves not only symptom management but also therapeutic relationships between patients and clinicians. Although discussions regarding the possibility of sentience in AI are increasing, the prevailing consensus is that AI lacks the capacity for authentic empathy, and the absence of dynamics such as transference and countertransference risks rendering relationships artificial and superficial (24). Sedlakova suggested that AI is better suited as an educational tool or data resource than a digital therapist, because human-like features may create misleading expectations.

#### 1.1.8 Algorithmic bias

AI systems risk perpetuating existing biases in their training data, potentially worsening inequalities for disadvantaged groups (25). Unsupervised algorithms may further amplify these biases, and mismatched objectives between AI systems and therapeutic goals can pose severe risks (26).

#### 1.1.9 Lack of ethical guidelines and normative uncertainty

As AI in psychiatry is a rapidly developing field, legal and ethical frameworks often lag behind technological progress (27). This gap may be filled with reactive policies only after harm has occurred (28). Currently, the absence of mental health–specific guidelines limits the safe and effective integration of AI systems (13, 25).

## 2 Methodology

### 2.1 Literature review

A literature search was conducted across PubMed/MEDLINE, Scopus, and Web of Science databases, covering publications up to early 2026. Relevant studies were identified using search terms related to artificial intelligence, psychiatry, mental health, medical ethics, digital health, and chatbots. Given the broad and evolving nature of ethical discussions surrounding AI-supported mental health technologies, a narrative literature review was deliberately chosen. This approach was considered more appropriate for the exploratory and conceptual aims of the study, as it allowed the integration of diverse perspectives, emerging concerns, and key ethical debates across a rapidly evolving field. The reviewed literature was used to identify recurring ethical themes and establish the conceptual foundation of the study, with particular attention to the core principles of biomedical ethics and key psychiatric ethical norms.

### 2.2 Analysis of AI applications

Given the evolving nature of this field, a brief clarification regarding terminology is warranted. The literature does not offer a clear or consistently applied distinction among terms such as “AI-supported mental health applications,” “mental health applications,”, “digital mental health tools” and “psychiatric applications,” which are often used interchangeably. In the present study, we use the term “AI-supported mental health applications” as a broad umbrella term encompassing AI-enabled mental health applications, chatbot-based support systems, and related digital tools. We considered this terminology the most appropriate because it captures the wide range of technologies examined while avoiding any implication that these applications constitute clinically validated psychiatric interventions.

Importantly, the applications examined in this study should not be understood as clinically validated diagnostic or treatment tools. Rather, they are AI-supported mental health applications primarily designed for purposes such as self-help, psychoeducation, symptom monitoring, emotional support, and complementary assistance. While some applications incorporate elements derived from evidence-based therapeutic approaches, they do not replace professional psychiatric assessment, diagnosis, or treatment.

Several widely used AI-supported mental health applications (including Wysa, Youper, Amaha, and Yuna) were examined. The evaluation considered technical, clinical, and ethical aspects such as data protection, fairness of access, responsibility and liability, transparency, consent practices, and potential impact on therapeutic interactions (See **Tables 1**, **2**). Applications were selected based on practical accessibility criteria, including being publicly available, downloadable, having substantial download numbers, media visibility, app-store presence, and relevance to psychiatric support functions.

**Table 1:** App-by-app ethical and clinical comparison of AI-supported mental health applications. .

Category	Question	Yuna	Wysa	Amaha	Youper
1. Data Privacy and Security	1.1 Standards for processing and storing mental health data?	EU General Data Protection Regulation	Amazon Web Services	Only noted as “protected”	—
	1.2 Can data be shared with third parties without user consent?	Providers disclosed the possibility of third-party data sharing in their privacy policies, although the specific circumstances, recipients, and limitations were not uniformly described.	Providers disclosed the possibility of third-party data sharing in their privacy policies, although the specific circumstances, recipients, and limitations were not uniformly described.	Providers disclosed the possibility of third-party data sharing in their privacy policies, although the specific circumstances, recipients, and limitations were not uniformly described.	Providers disclosed the possibility of third-party data sharing in their privacy policies, although the specific circumstances, recipients, and limitations were not uniformly described.
	1.3 Is the privacy policy easily understandable and accessible?	Yes	Yes	Yes	Yes
2. Fairness and Access Equality	2.1 Do algorithms produce disadvantageous outcomes for demographic groups?	No publicly available evidence of demographic bias was identified; however, independent bias audits were not publicly disclosed.	No publicly available evidence of demographic bias was identified; however, independent bias audits were not publicly disclosed.	No publicly available evidence of demographic bias was identified; however, independent bias audits were not publicly disclosed.	No publicly available evidence of demographic bias was identified; however, independent bias audits were not publicly disclosed.
	2.2 Are free or low-cost alternatives offered to reduce economic inequalities?	Most applications offered free or low-cost access to at least some features, although advanced functions often required paid subscriptions.	Most applications offered free or low-cost access to at least some features, although advanced functions often required paid subscriptions.	Most applications offered free or low-cost access to at least some features, although advanced functions often required paid subscriptions.	Most applications offered free or low-cost access to at least some features, although advanced functions often required paid subscriptions.
3. Transparency and Accountability	3.1 Can the user understand how the AI’s decisions are reached?	Decision-making mechanisms are not fully explained to users.	Decision-making mechanisms are not fully explained to users.	Decision-making mechanisms are not fully explained to users.	Decision-making mechanisms are not fully explained to users.
	3.2 Who bears responsibility for incorrect suggestions or outcomes?	Responsibility is not clearly defined. Terms of service generally place substantial responsibility on users while limiting provider liability.	Responsibility is not clearly defined. Terms of service generally place substantial responsibility on users while limiting provider liability.	Responsibility is not clearly defined. Terms of service generally place substantial responsibility on users while limiting provider liability.	Responsibility is not clearly defined. Terms of service generally place substantial responsibility on users while limiting provider liability.
	3.3 Is the data used in algorithm training publicly available?	Information regarding training datasets was not publicly disclosed.	Information regarding training datasets was not publicly disclosed.	Information regarding training datasets was not publicly disclosed.	Information regarding training datasets was not publicly disclosed.
4. User Consent	4.1 Does the app provide sufficient information about risks and limitations?	Partially provides explicit information regarding potential risks and limitations	No standardized disclosure of risks and limitations was observed.	No standardized disclosure of risks and limitations was observed.	No standardized disclosure of risks and limitations was observed.
	4.2 Is the language used in the consent process clear and understandable?	Consent language was generally understandable	Consent language was generally understandable	Consent language was generally understandable	Consent language was generally understandable
5. Psychological Impact and Do No Harm	5.1 Can the app cause emotional harm through misdirection or incorrect outcomes?	Potential emotional harm cannot be excluded, particularly in situations involving inaccurate, inappropriate, or misleading responses.	Potential emotional harm cannot be excluded, particularly in situations involving inaccurate, inappropriate, or misleading responses.	Potential emotional harm cannot be excluded, particularly in situations involving inaccurate, inappropriate, or misleading responses.	Potential emotional harm cannot be excluded, particularly in situations involving inaccurate, inappropriate, or misleading responses.
	5.2 Can a self-help model increase feelings of loneliness or isolation?	Self-help models may reduce opportunities for human interaction and could contribute to feelings of isolation in some users.	Self-help models may reduce opportunities for human interaction and could contribute to feelings of isolation in some users.	Self-help models may reduce opportunities for human interaction and could contribute to feelings of isolation in some users.	Self-help models may reduce opportunities for human interaction and could contribute to feelings of isolation in some users.
6. Clinical Application and Diagnosis	6.1 What parameters does the algorithm consider when diagnosing?	Does not diagnose	Does not diagnose	Does not diagnose	Does not diagnose
	6.2 How are false positive/negative results managed?	Does not diagnose	Does not diagnose	Does not diagnose	Does not diagnose
	6.3 Does it have scientific validity?	Reported use of CBT and mindfulness-based techniques	Reported use of CBT and mindfulness-based techniques	Reported use of CBT and mindfulness-based techniques	Yes, but not specified
7. Treatment Plans	7.1 Are recommendations personalized or generalized?	Recommendations were generated using user-provided information, with varying degrees of personalization.	Recommendations appeared to be largely standardized, with limited adaptation based on user-provided information.	No	Partially
	7.2 Are living conditions and environmental factors considered?	Consideration of environmental and social context appeared limited and largely depended on information voluntarily provided by users.	No	No	No
8. Empathy and Relationship Dynamics	8.1 What tools does the app use to mimic therapists’ empathic approach?	Applications employed supportive and empathic conversational language, although emotional depth remained limited.	Applications employed supportive and empathic conversational language, although emotional depth remained limited.	Applications employed supportive and empathic conversational language, although emotional depth remained limited.	Applications employed supportive and empathic conversational language, although emotional depth remained limited.
	8.2 What alternative does AI offer to the patient-therapist relationship?	Functions as a supplementary support tool; but lacks the depth, continuity, and human presence that define a genuine therapeutic relationship.	Functions as a supplementary support tool; but lacks the depth, continuity, and human presence that define a genuine therapeutic relationship.	Functions as a supplementary support tool; but lacks the depth, continuity, and human presence that define a genuine therapeutic relationship.	Functions as a supplementary support tool; but lacks the depth, continuity, and human presence that define a genuine therapeutic relationship.
	8.3 Are users inclined to form emotional bonds with the app?	Applications are designed to simulate supportive interactions, which may facilitate emotional attachment in some users.	Applications are designed to simulate supportive interactions, which may facilitate emotional attachment in some users.	Applications are designed to simulate supportive interactions, which may facilitate emotional attachment in some users.	Applications are designed to simulate supportive interactions, which may facilitate emotional attachment in some users.
9. Acute Situations	9.1 Can it detect suicide risk or violent tendencies?	No	Crisis-related responses were observed during user testing, suggesting some level of risk-detection functionality.	No	Crisis-related responses were observed during user testing, suggesting some level of risk-detection functionality.
	9.2 Is there an emergency intervention mechanism?	No	The application provided crisis resources and referral information for emergency situations.	No	The application provided crisis resources and referral information for emergency situations.
10. Clinical Accuracy	10.1 Which clinical guidelines does it follow?	References CBT-based approaches; information regarding guideline updates was unavailable.	References CBT-based approaches; information regarding guideline updates was unavailable.	References CBT-based approaches; information regarding guideline updates was unavailable.	References CBT-based approaches; information regarding guideline updates was unavailable.
	10.2 How effective is it compared to traditional therapy?	Current evidence suggests these tools may complement traditional therapy, particularly for mild-to-moderate symptoms, but should not be considered equivalent replacements.	Current evidence suggests these tools may complement traditional therapy, particularly for mild-to-moderate symptoms, but should not be considered equivalent replacements.	Current evidence suggests these tools may complement traditional therapy, particularly for mild-to-moderate symptoms, but should not be considered equivalent replacements.	Current evidence suggests these tools may complement traditional therapy, particularly for mild-to-moderate symptoms, but should not be considered equivalent replacements.
11. Communication Style	11.1 How is the language designed to build user trust? (Formal/Friendly/Neutral)	Generally employed supportive, friendly, and non-judgmental communication styles.	generally employed supportive, friendly, and non-judgmental communication styles.	generally employed supportive, friendly, and non-judgmental communication styles.	generally employed supportive, friendly, and non-judgmental communication styles.
	11.2 Can the conversational tone create psychological pressure?	The psychological impact of conversational tone may vary among users and has not been systematically evaluated across applications.	The psychological impact of conversational tone may vary among users and has not been systematically evaluated across applications.	The psychological impact of conversational tone may vary among users and has not been systematically evaluated across applications.	The psychological impact of conversational tone may vary among users and has not been systematically evaluated across applications.
12. Normalization of Concepts	12.1 Are psychiatric concepts used medically or in popular terms?	Psychiatric concepts were generally presented using simplified, evidence-informed mental health terminology.	Psychiatric concepts were generally presented in popular, user-friendly language rather than strictly clinical terminology.	Psychiatric concepts were generally presented using simplified, evidence-informed mental health terminology.	Psychiatric concepts were generally presented using simplified, evidence-informed mental health terminology.
13. Social Impacts	13.1 Does the app reduce stigma by spreading mental health services, or does it create digital dependency?	Applications may improve access to mental health support and help reduce stigma by providing a private and accessible means of seeking support, while also raising concerns regarding excessive reliance on digital systems.	Applications may improve access to mental health support and help reduce stigma by providing a private and accessible means of seeking support, while also raising concerns regarding excessive reliance on digital systems.	Applications may improve access to mental health support and help reduce stigma by providing a private and accessible means of seeking support, while also raising concerns regarding excessive reliance on digital systems.	Applications may improve access to mental health support and help reduce stigma by providing a private and accessible means of seeking support, while also raising concerns regarding excessive reliance on digital systems.
	13.2 How does AI’s widespread use affect psychiatrists’ workload?	AI tools may assist with selected tasks such as psychoeducation and monitoring; evidence regarding overall workload reduction remains limited.	AI tools may assist with selected tasks such as psychoeducation and monitoring; evidence regarding overall workload reduction remains limited.	AI tools may assist with selected tasks such as psychoeducation and monitoring; evidence regarding overall workload reduction remains limited.	AI tools may assist with selected tasks such as psychoeducation and monitoring; evidence regarding overall workload reduction remains limited.
14. Technology and Human Interaction	14.1 Can AI apps completely replace the need for face-to-face therapy?	Current evidence supports the use of AI applications as complementary tools rather than replacements for face-to-face psychiatric care.	Current evidence supports the use of AI applications as complementary tools rather than replacements for face-to-face psychiatric care.	Current evidence supports the use of AI applications as complementary tools rather than replacements for face-to-face psychiatric care.	Current evidence supports the use of AI applications as complementary tools rather than replacements for face-to-face psychiatric care.

CBT, Cognitive Behavioral Therapy; RCT, Randomized Controlled Trial.

**Table 2:** General Ethical and Clinical Overview of AI-supported mental health applications.

Category	Questions	Answers
Data Privacy and Security	What standards does the application follow for processing and storing users’ mental health data?	Compliance with GDPR was reported in some applications; several applications indicated the use of cloud infrastructure (e.g., Amazon Web Services), while others provided limited publicly available information.
	Can data be shared with third parties without the user’s consent?	Data sharing practices appear to be subject to applicable legislation and privacy policies; the extent of third-party sharing varies across applications.
	Is the privacy policy understandable and accessible to users?	Privacy policies were generally accessible, although readability and comprehensibility may vary among users.
Justice and Equal Access	Do the application’s algorithms produce biased outcomes for certain demographic groups?	No publicly available evidence of demographic bias was identified during the assessment; however, the absence of evidence should not be interpreted as evidence of absence.
	Does it offer free or low-cost alternatives to reduce economic inequality?	Most applications provide free features or lower-cost options that may improve access to mental health support; however, free versions generally appear to offer fewer features and functionalities compared with paid versions.
Transparency and Accountability	Can users understand how AI makes its decisions?	Information regarding algorithmic decision-making was generally limited, making it difficult for users to fully understand how outputs are generated.
	Who is responsible in case of incorrect suggestions or outcomes?	Responsibility allocation appears to be primarily user-centered, based on available terms of service, while provider liability is often limited.
	Are the datasets used to train the algorithm publicly available?	Information regarding training datasets was not publicly disclosed.
User Consent	Does the application provide sufficient information about risks and limitations?	Information regarding risks and limitations was often limited or incompletely described in publicly available materials.
	Is the language used in the consent process clear and understandable?	Consent language was generally written in accessible terms, although comprehension may vary among users.
Psychological Impact and Non-Maleficence	Can the application cause emotional harm through misguidance or incorrect results?	No substantial evidence was identified during the assessment; however, the possibility of emotional harm cannot be excluded.
	Could a self-help model increase feelings of loneliness or isolation in users?	Self-help models may contribute to feelings of isolation in some users, although evidence remains limited and context-dependent.
Clinical Practice and Diagnosis	What parameters does the algorithm consider when making a diagnosis?	Assessed applications did not claim to provide formal psychiatric diagnoses.
	How are false positives/negatives managed?	Explicit information regarding the management of false positive or false negative outputs was generally not identified.
	Is there scientific validity?	Scientific support varied across applications, ranging from references to evidence-based approaches such as CBT to limited publicly available validation data.
Treatment Plans	Are the recommendations personalized or generalized?	Recommendations appear to be partially personalized based on user-provided information and interaction history.
	Are living conditions and environmental factors taken into account?	Consideration of environmental and contextual factors appears limited to the information voluntarily provided by users.
Empathy and Relationship Dynamics	What tools does the application use to mimic the empathetic approach of human therapists?	Applications commonly employ supportive and empathy-oriented communication styles, although users may perceive these interactions differently.
	How does AI offer an alternative to the patient-therapist relationship?	AI applications are generally presented as supportive communication tools rather than direct substitutes for therapeutic relationships.
	Do users tend to form emotional bonds with the application?	Emotional attachment to AI applications may develop in some users; however, publicly available evidence remains limited and appears to vary across individuals and contexts.
Acute Situations	Can it detect situations like suicide risk or violent tendencies?	Crisis detection capabilities appear limited and vary substantially across applications.
	Is there an emergency intervention mechanism?	Most applications do not provide direct emergency intervention; some offer guidance or referral to local support resources.
Clinical Accuracy	Which clinical guidelines does it rely on?	Several applications refer to CBT-based frameworks, although information regarding guideline updates and maintenance is often limited.
	How effective is it compared to traditional therapy?	Direct comparisons with traditional therapy remain limited and may vary according to application design and clinical context.
Communication Style	How is the application’s language designed to build user trust? (Formal, friendly, neutral)	Communication styles were generally designed to be friendly and non-judgmental; perceived authenticity may vary among users.
	Can the tone used during conversations create psychological pressure?	Certain communication styles may be perceived as psychologically influential by some users, although evidence remains limited.
Normalization of Concepts	Are psychiatric concepts used in a medical or popularized way?	Psychiatric concepts were generally presented using terminology derived from evidence-based psychotherapy and mental health frameworks.
Social Impacts	Does the application help reduce stigma by expanding access to mental health services, or does it create a new form of digital dependency?	These applications may reduce barriers to mental health support while also raising concerns regarding potential dependency in some users.
	How does the spread of AI affect the workload of psychiatric professionals?	AI-supported tools may contribute to reducing workload in selected contexts, although empirical evidence remains limited.
Technology and Human Interaction	Can AI applications eliminate the need for face-to-face therapy?	Current evidence and application design generally position these tools as complementary to, rather than replacements for, face-to-face therapy.

Information presented in these tables are derived from publicly available information provided by the application developers, direct assessment of the applications by the authors, or the authors’ interpretation where explicit information was not available.

The application analysis was conducted between April 2025 and November 2025. Applications were accessed through the App Store in Türkiye. The evaluated versions included Wysa (3.8.4), Youper (6.06.006), Amaha (3.109), and Yuna (1.0.143). Both free and paid versions were reviewed. Test accounts were created using fictitious user information, and no real patient data were entered.

To improve the reproducibility of the research, we clarified the user scenarios used during the assessment, including a standardized suicide-risk scenario in which the user expressed suicidal thoughts and emotional distress. Privacy and data protection were evaluated by reviewing privacy policies, terms of service, and data-sharing disclosures. Informed consent procedures were assessed by examining onboarding processes, consent forms, and user information materials. Crisis-response mechanisms were evaluated by exploring how applications responded to scenarios involving suicidal ideation, self-harm, or psychological emergencies. Accountability and transparency features were assessed based on the availability of information regarding decision-making processes, responsibility for outcomes, and disclosures concerning AI functionality.

The analyzed applications in this study were publicly available consumer-facing tools, and the evaluation relied exclusively on publicly accessible information, user interfaces, privacy policies, and terms of service. No proprietary data, restricted-access materials, or developer resources were used; therefore, no formal permission from application developers was required.

### 2.3 Clinician interviews

To complement the documentary analysis, five psychiatrists were interviewed using a semi-structured format. All five were practicing psychiatrists based in Turkey, with a minimum of five years of clinical experience, working in either university-affiliated or public hospital settings at the time of the interviews (See **Table 3**), and all interviews were conducted remotely via video call. Psychiatrists were recruited using snowball sampling. Interviews continued until enough depth was reached, with later interviews mostly confirming earlier themes rather than bringing up new ideas. None of the participants were involved in the development of any AI application. All participants were informed of the study’s purpose, voluntary nature, and their right to withdraw at any time without consequence. Both verbal and written informed consent were obtained from each participant before data collection began. To protect anonymity, participants are referred to throughout this paper using assigned codes (P1–P5).

**Table 3:** Participant characteristics.

Participant	Gender	Age	Setting	Years of experience	Prior AI tool use
P1	Female	41	University Hospital	10 years	None
P2	Male	42	University Hospital	11 years	Moderate (clinical context)
P3	Male	38	University Hospital	10 years	Limited (personal use)
P4	Male	36	Public Psychiatric Clinic	9 years	Moderate (clinical context)
P5	Male	40	Public Psychiatric Clinic	9 years	None

The interviews were conducted by a medical doctor with academic interests in psychiatry and bioethics. Throughout the interviews and data analysis, the researcher maintained reflective notes and regularly discussed emerging findings and interpretations with academic supervisor to promote reflexivity and minimize the influence of individual assumptions. The researcher had no supervisory, hierarchical, or dependent relationship with any of the participants.

The interviews were conducted in Turkish using a semi-structured format and lasted approximately 20–30 minutes. Audio recordings were transcribed verbatim by the researchers. An inductive coding strategy inspired by grounded theory was employed. Initial open codes were generated from the transcripts, followed by the development of broader conceptual categories through iterative comparison. To improve analytical rigor, emerging themes were repeatedly compared across interviews and discussed within the research team.

Grounded theory techniques were used to identify themes and generate conceptual insights that could be compared with the findings of the literature review and application analysis. Grounded theory was selected because the field lacks an established explanatory framework, and the study aimed to generate a conceptual model of emerging ethical issues from participant narratives. All transcripts were coded by the primary researcher. Although formal second coding was not performed, theme development and coding decisions were discussed iteratively with academic supervisor to strengthen interpretation and reduce individual bias. Participant checking was not conducted.

### 2.4 Ethical evaluation framework

The empirical material was analyzed through a dual-layer approach.

#### 2.4.1 Layer one: coherence perspective

The first layer of the framework evaluates whether AI-supported mental health applications are normatively coherent with the ethical foundations of psychiatric practice. In this context, coherence refers to the degree of compatibility between the functioning of an AI-supported intervention and the established ethical norms that have traditionally guided psychiatric care.

Unlike general medical practice, psychiatry relies heavily on relational and contextual ethical obligations. Therefore, this layer focuses not only on biomedical principles but also on psychiatric ethics norms that have developed through clinical practice. These include confidentiality and privacy, meaningful informed consent, respect for patient autonomy, preservation of the therapeutic relationship, professional responsibility, trustworthiness, and protection of vulnerable individuals.

Applications and interview findings were assessed according to whether they supported, undermined, or created tensions with these established norms. For example, opaque data-processing practices may conflict with confidentiality and informed consent requirements, while limited crisis-response mechanisms may challenge professional responsibilities toward vulnerable patients.

This coherence-based assessment differs from many existing AI ethics frameworks, which primarily focus on technical principles such as transparency, fairness, explainability, robustness, and accountability. While these dimensions remain important, the present framework emphasizes whether AI systems can function within the distinctive ethical environment of psychiatric care, where trust, therapeutic alliance, empathy, and contextual understanding play central roles. In this sense, the framework evaluates not only technological performance but also normative compatibility with psychiatric practice.

#### 2.4.2 Layer two: principle-based perspective

Following the coherence assessment, ethical issues were further analyzed using Beauchamp and Childress’s four principles of biomedical ethics: autonomy, beneficence, non-maleficence, and justice. This second layer was designed to provide a structured method for evaluating the practical ethical implications of AI-supported mental health applications.

Each identified ethical concern was examined according to its potential impact on one or more principles. For example, inadequate disclosure of algorithmic processes and data use practices was analyzed primarily in relation to autonomy, whereas privacy breaches and insufficient crisis-response mechanisms were evaluated through the principle of non-maleficence. Questions regarding accessibility, affordability, and algorithmic bias were examined through the principle of justice, while claims concerning symptom improvement, treatment support, or increased access to care were assessed in relation to beneficence.

In cases where principles appeared to conflict, the analysis did not assume that one principle automatically overrides another. Instead, ethical tensions were explicitly identified and discussed. For example, increasing accessibility through automated systems may promote beneficence and justice while simultaneously creating risks for autonomy, privacy, or therapeutic integrity. Likewise, extensive data collection may improve personalization and potential clinical benefit but may also threaten confidentiality and informed consent.

This principle-based layer complements the coherence perspective by translating broader psychiatric ethical concerns into a structured framework for ethical evaluation. Together, the two layers allow both normative compatibility and practical ethical consequences to be examined simultaneously.

### 2.5 Rationale for the two-tier approach

This framework was designed to strengthen the analysis in two ways. The coherence perspective is rooted in discussions of established psychiatric traditions, whereas the principle-based perspective provides a more pragmatic lens for assessing concrete dilemmas. Together, they allow for a comprehensive ethical evaluation of AI-supported psychiatry that is both theoretically grounded and practically oriented.

To improve the trustworthiness of the study, findings were examined through three different sources: literature review, app analysis, and semi-structured interviews. Notes were kept throughout the analysis process, and interpretations were regularly discussed with supervisors to support balanced conclusions.

## 3 Results

### 3.1 Part I: evaluation of AI applications

Four AI-supported mental health applications (Wysa, Youper, Amaha, and Yuna) were analyzed across ethical, technical, and clinical dimensions under 14 headings. Key themes included the following:

- Data Privacy and Security: All applications implemented protective measures, but only Yuna explicitly referenced the GDPR. Transparency regarding data sharing was limited, and training datasets remained undisclosed.
- Justice and Equal Access: Applications offered generalized support intended to prevent demographic discrimination, but external audits for bias were limited. Access to full services often required payment.
- Transparency and Accountability: AI decision-making processes were not disclosed to users, and liability was largely shifted to users in cases of misleading guidance.
- Consent and Information: Consent mechanisms were present, but disclosures regarding risks were often embedded within the applications. Only Yuna provided clear information during the setup process.
- Psychological Impact and Non-Maleficence: Applications avoided direct intervention or diagnosis but might provide insufficient support for severe cases. Self-help models may also weaken human relational bonds.
- Clinical Validity and Diagnosis: None of the applications provided formal diagnoses. The recommendations were based on CBT, mindfulness, or positive psychology, with varying levels of external validation.
- Empathy and Relational Dynamics: Empathic responses were often generic, although superficial emotional connections could still be formed.
- Emergency Response: Crisis detection was limited, and support typically involved referral to local hotlines.
- Clinical Accuracy and Effectiveness: Applications may complement, but not replace, therapy and appear most suitable for mild-to-moderate conditions.
- Language and Communication: Communication styles were friendly and non-judgmental, but generic responses could appear artificial or superficial.
- Societal Impact: Although greater accessibility may help reduce stigma, there remains a risk of excessive dependence on digital tools.
- Technology and Human Interaction: AI-supported mental health applications are best understood as complementary tools rather than replacements for clinicians. They can widen access to support, offer immediate availability, and assist with symptom monitoring, but they lack the genuine empathy, contextual judgment, and interpersonal understanding central to psychiatric care.

### 3.2 Part II: psychiatrists’ perspectives

#### 3.2.1 Interview questions

1. In your opinion, what is the likelihood that future technologies or artificial intelligence could perform the tasks of an average psychiatrist as well as or even better than human physicians? Why?
2. What do you think is the likelihood that future technologies or artificial intelligence will replace human physicians in performing specific psychiatric interventions?
3. In the coming years, what kind of changes do you think artificial intelligence will bring to psychiatric practice? Can you explain this point briefly?
4. What do you think are the potential benefits of AI-supported mental health applications in clinical practice?
5. What do you think are the potential risks or harms associated with AI-supported mental health applications in clinical practice?
6. What ethical issues may arise from the use of AI-supported mental health applications in clinical settings?
7. How does the use of AI-supported mental health applications affect the ethical challenges that already exist in psychiatry as a discipline?
8. Do you feel prepared as a psychiatrist to address the risks and ethical challenges that artificial intelligence may introduce?
9. Do you think young psychiatrists are receiving sufficient training or preparation regarding these issues?
10. Are you aware of the use of artificial intelligence in clinical psychiatric practice? Have you used AI-based tools or applications?

Interviews with psychiatrists provided deeper insight into how AI-supported applications are perceived in clinical practice. Four main themes emerged from the grounded theory analysis.

##### 3.2.1.1 Digital empathy limitations

Clinicians consistently emphasized that conversational agents can only simulate empathy at a surface level. Although they may generate phrases that appear supportive, they lack the genuine emotional understanding and relational depth required for psychiatric care. This limitation was viewed as particularly problematic for the therapeutic relationship, which relies heavily on authentic empathy and trust. As one participant noted, “AI can imitate empathy, but it cannot truly build a therapeutic bond the way we can.” (P2).

##### 3.2.1.2 Clinical role displacement

Another common concern was that AI tools, which are intended to support care, could gradually replace genuine clinical interactions. Psychiatrists worry that, as healthcare systems face pressure to reduce costs and manage large numbers of patients, they may increasingly rely on AI, especially for mild-to-moderate cases. This could reduce the role of clinicians and weaken the value of human expertise. One participant highlighted the economic dimension of this risk, stating, “For many patients, regular therapy is expensive, so something introduced as support could slowly take the place of real human care.” (P5) Another participant noted that it remains unclear who is responsible when harm is done: “The question is not whether AI can assist — of course it can. The question is who remains responsible when something goes wrong. Right now, that answer is unclear.” (P3).

##### 3.2.1.3 Emotional responses to AI

Interviewees also reflected on the ambivalence that patients and clinicians might experience toward these technologies. Patients may find chatbots non-judgmental and approachable, which could encourage the disclosure of sensitive information. Conversely, this sense of neutrality may create a false feeling of safety and draw individuals away from genuine human connection, thereby increasing isolation. Clinicians themselves reported mixed feelings, ranging from cautious optimism regarding new opportunities to deep skepticism about the long-term impacts on psychiatric practice. As one clinician explained, “Some patients may feel safer with AI at first, but without human connection it can become another form of loneliness.” (P1).

##### 3.2.1.4 Ontological mismatch with psychiatry

A broader theme concerned the fundamental differences between psychiatry and AI systems. Psychiatrists noted that their discipline extends beyond symptom management to include subjective experiences, contextual meanings, and human relationships. They worried that, by focusing narrowly on data and patterns, AI tools risk neglecting the existential and relational dimensions of psychiatry. Reflecting this concern, one participant noted, “Psychiatry is not just treating symptoms; it is understanding the person, their relationships, and the meaning of what they are going through.” (P4).

#### Conceptual map

**Figure 1:**
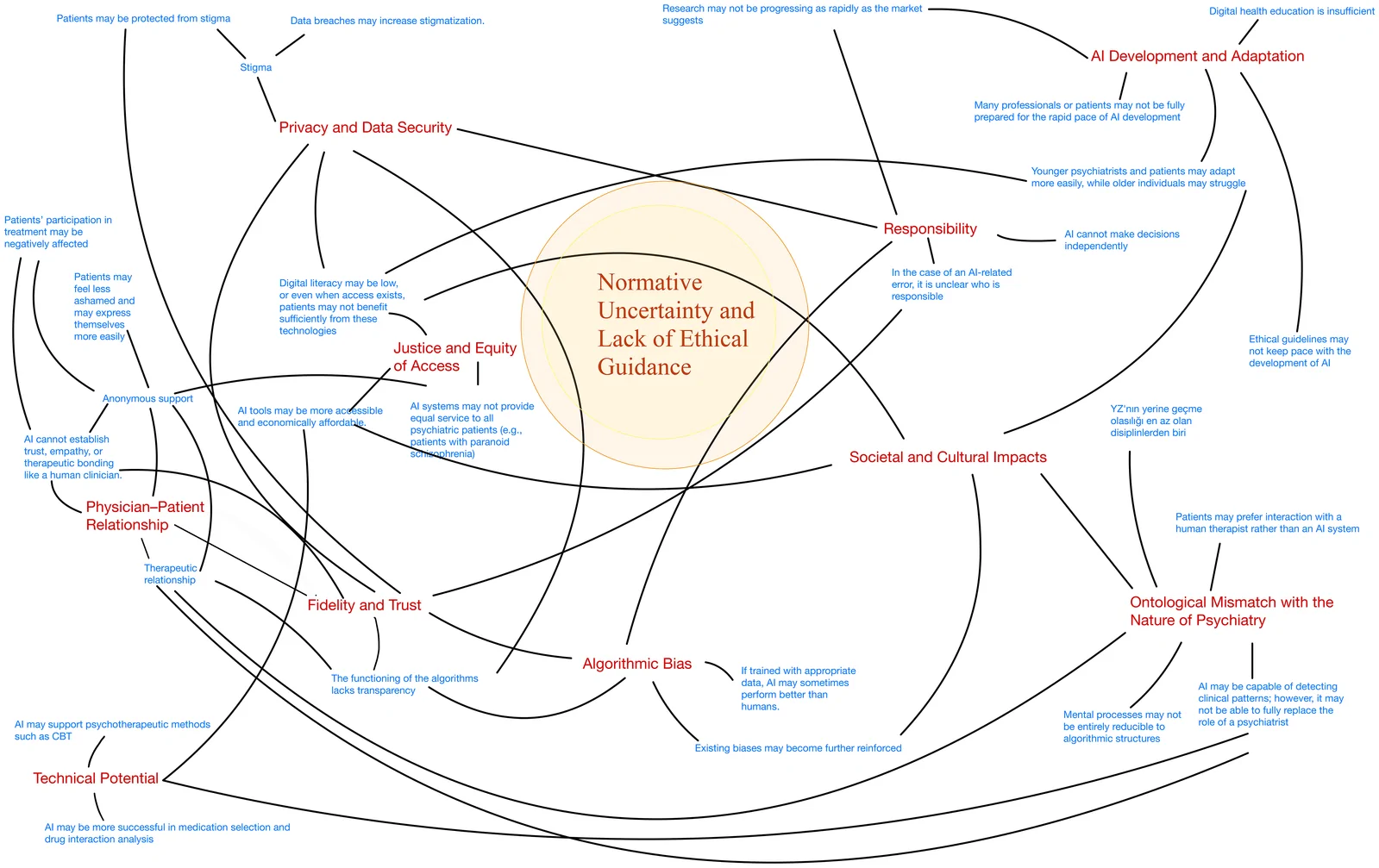
Conceptual map of the ethical framework developed in this study. The framework emerged through the integration of three complementary sources of evidence: the narrative literature review, the structured analysis of AI-supported mental health applications, and qualitative interviews with psychiatrists. Interview data were analyzed using grounded theory techniques to generate codes, conceptual categories, and overarching themes, which were iteratively compared with findings from the literature review and application analysis. Areas of convergence across these data sources informed the development of the ethical domains presented in the figure. These domains were subsequently organized within the study’s two-layer analytical framework, comprising the Coherence Perspective and the Principle-Based Perspective, to illustrate both their normative compatibility with psychiatric ethics and their practical implications within the principles of biomedical ethics.

These themes were integrated with the findings from the literature review and application analysis into a conceptual map (See **Figure 1**). The map demonstrates how the identified ethical tensions work across three levels.

1. Normative level – coherence or conflict with established psychiatric ethics;
2. Practical level – clinical role, data protection, and relational limitations;
3. Perceptual level – clinicians’ emotional and professional reactions to AI.

## Discussion

The findings of the current study highlight both the potential benefits and risks of using AI-supported tools in psychiatric practice. These technologies can extend access to care, reduce barriers created by stigma, and provide continuous support to individuals with mild-to-moderate mental health concerns (29, 30). However, they also raise important concerns regarding privacy, transparency, responsibility, and the potential erosion of the therapeutic relationship (31, 32). Although these tensions are not new in psychiatry, digital innovation has changed its scale and pace (33).

For example, suicide cases associated with AI chatbot use, some of which have already reached legislative hearings in the United States, demonstrate the potentially harmful and even fatal consequences of inadequate ethical oversight. Improperly designed AI systems may lead to misclassification, inappropriate interventions, and even an increased risk of suicide due to stigma, legal consequences, or mismanagement of sensitive data (34). These risks highlight that alignment with psychiatric norms and adherence to core ethical principles such as autonomy, privacy, and informed consent are not merely abstract concepts, but have real consequences for patient safety (34).

By applying a dual-layer ethical framework, the current study offers a way to examine challenges beyond existing discussions. From a coherence-based ethical perspective, AI applications in mental healthcare reveal significant tensions with long-established psychiatric norms, particularly regarding confidentiality, accountability, and the provision of meaningful informed consent. While traditional psychiatric practice is grounded in strict obligations to protect patient privacy and ensure that consent is both informed and contextually meaningful, AI systems frequently operate through opaque data-processing mechanisms, often without explicit or adequately understood patient authorization (34). Moreover, current AI governance frameworks are underdeveloped, with unclear responsibilities, limited regulatory oversight, and inconsistent use of safety measures such as crisis escalation mechanisms (35). These limitations not only raise ethical concerns in psychiatric care but also increase the risk of misclassification, inappropriate interventions, and potential harm. This demonstrates that alignment with core ethical principles is not merely theoretical, but essential for protecting patient wellbeing in AI-supported care.

The principle-based analysis added an additional layer by linking real-world issues to the four core ethical principles: autonomy, beneficence, non-maleficence, and justice. For example, concerns regarding data privacy, limited explainability, and algorithmic bias may undermine autonomy and justice. Concurrently, risks such as incorrect outputs or inadequate clinical responses challenge beneficence and non-maleficence (31, 32). This two-step approach helps clarify not only where AI aligns or fails to align with psychiatry, but also how it may affect patients in everyday clinical practice.

These insights contribute to the expanding literature on digital mental health, which has raised similar concerns (32, 36), particularly regarding data protection, algorithmic bias, and the spread of misinformation associated with generative AI systems. However, most studies have examined ethical principles and psychiatric norms separately. By bringing them together, our current research demonstrates the value of combining theoretical and practical perspectives. This layered approach helps identify important nuances that may be overlooked when relying on a single framework.

The ethical concerns identified in this study are also reflected in contemporary AI governance frameworks. Both the WHO Guidance on Ethics and Governance of Artificial Intelligence for Health (37) and the UNESCO Recommendation on the Ethics of Artificial Intelligence (38) emphasize the need for trustworthy, human-centered AI through principles such as transparency, accountability, fairness, and respect for human rights. The consistency between these international recommendations and the concerns expressed by participating psychiatrists supports the relevance of the ethical framework proposed in this study.

The present study has several limitations. The application analysis was limited to publicly accessible information, including user interfaces, privacy policies, terms of use, and app responses to a standardized user scenario; additionally, access to some applications was restricted in Türkiye. We did not have access to the underlying algorithms, training data, internal decision-making processes, or other backend features of the applications, nor were developer perspectives available. Consequently, our evaluation of ethical dimensions such as transparency, accountability, and crisis response capabilities was necessarily based on externally observable characteristics rather than the applications’ internal functioning. Therefore, our assessments were limited to externally observable features and should be interpreted as indicative rather than definitive evaluations of the underlying systems. The interviews involved only five psychiatrists, all recruited through snowball sampling within the Turkish healthcare context, which limits the transferability of the findings to other clinical settings, healthcare systems, and digital platforms. Cultural and systemic characteristics specific to Türkiye may also have influenced the emergence and interpretation of themes such as ontological mismatch, clinical role displacement, digital empathy, and clinicians’ emotional responses toward AI-supported mental health tools. Moreover, AI technologies are evolving rapidly; therefore, ethical evaluations are likely to change as new applications emerge (35). Future research should expand the scope by including larger clinician samples, exploring patients’ perspectives, and assessing the role of developers and technology companies in shaping ethical outcomes. Further real-world and longitudinal studies are also required to evaluate the long-term safety, effectiveness, and clinical integration of AI systems in psychiatry (39).

An additional limitation relates to the assessment of clinical validity and suicide-related safety claims in AI-supported mental health applications. Although some applications report crisis-detection capabilities and employ evidence-based approaches, independent evidence regarding their clinical effectiveness, reliability, and performance in high-risk situations remains limited. Consequently, conclusions regarding their ability to detect suicide risk, prevent self-harm, or provide clinically meaningful interventions should be interpreted with caution. Larger-scale clinical studies, independent validation research, and longitudinal evaluations are needed to better understand the effectiveness and safety of these technologies across diverse populations and clinical contexts. Nevertheless, continued advances in artificial intelligence, together with improvements in clinical validation and safety mechanisms, may contribute to the development of more reliable and effective tools for supporting mental healthcare in the future.

Finally, this study did not include the perspectives of patients, caregivers, family members, individuals with impaired decision-making capacity, or application developers. As a result, the ethical analysis primarily reflects clinicians’ perspectives and may not fully capture how AI-supported mental health technologies are experienced or designed in practice. For example, patients may perceive digital empathy, trust, and therapeutic interactions differently from clinicians, while developers could provide valuable insight into the technical constraints and design decisions underlying these applications. Incorporating these perspectives would contribute to a more comprehensive and balanced ethical framework for AI-supported mental health technologies.

The variability observed in app responses, particularly regarding crisis management, illustrates the value of the proposed dual-layer framework. Although these differences can be evaluated at the practical level through principles such as non-maleficence, beneficence, and justice, they also raise a broader normative question of whether AI-supported mental health applications are ethically coherent with the relational responsibilities, duty of care, and therapeutic obligations that define psychiatric practice. These findings further illustrate the value of evaluating AI-supported mental health applications through both layers of the proposed framework.

Taken together, these reflections suggest that the ethical integration of AI into psychiatry requires more than technical innovation; it requires sustained attention to long-standing professional values and a commitment to protecting the rights and wellbeing of patients.

## Conclusion

Beyond offering a new methodological lens, the current study also suggests concrete steps for integrating AI into psychiatry in a safer and more responsible manner. First, there is a need for greater oversight. These applications involve some of the most sensitive forms of personal data; clear protections, independent audits, and well-defined responsibilities must be established if harm occurs. Patients, clinicians, and developers should not be left uncertain about who is accountable.

Second, informed consent must be redesigned for use in the digital environment. Currently, patients are often asked to agree to lengthy and complex technical documents. Instead, consent processes should be clear and accessible, giving patients a genuine choice regarding how their information is used, how the technology operates, and what its limitations are.

Third, these tools must be fairly tested and properly maintained. Many applications have never been evaluated in rigorous clinical studies, and there is still insufficient transparency regarding how they are programmed. Without such safeguards, there is a risk of hidden bias that may disadvantage the groups most in need of care. Independent validation, regular bias assessments, and greater openness regarding data and algorithms are essential if these technologies are to be trusted.

Fourth, as AI technologies continue to evolve, regulatory and ethical guidance will play an increasingly important role in shaping their use in mental healthcare. Future policies should ensure that AI-supported mental health applications are developed and implemented in ways that remain consistent with both established psychiatric values and emerging international standards for trustworthy and human-centered AI.

Finally, clinicians should remain at the center of psychiatric care. AI can assist by supporting patients, expanding access, and easing pressure on healthcare systems; however, given the current level of technological readiness, AI cannot replace the empathy, judgment, and therapeutic bond that arise from human interaction. Therefore, these tools should be used to complement clinical practice rather than replace it, and psychiatrists should play a leading role in how they are introduced and applied.

The ethical questions surrounding AI extend beyond technological performance. They concern how systems are designed, governed, and implemented in clinical practice. When these considerations are addressed appropriately, AI has the potential to become a valuable complement to mental healthcare; when they are overlooked, it may undermine the ethical values on which psychiatric practice depends.

More broadly, the growing role of AI in psychiatry reflects an evolving relationship between technology and human experience. As AI systems increasingly simulate human interaction, ethical evaluation must extend beyond issues of safety and effectiveness to include trust, authenticity, and the preservation of genuinely human relationships in care.

Accordingly, stronger regulatory frameworks are needed to keep pace with advances in AI-supported mental healthcare. Clear standards for transparency, accountability, clinical validation, and data protection will be essential to ensure that technological progress remains aligned with the ethical foundations of psychiatric practice.

## Considerations

Existing literature has primarily examined AI in psychiatry from the perspectives of patients and medical ethics. However, these applications are developed by software engineers, whose awareness of and sensitivity to ethical principles remain unclear. Future studies should critically examine how developers and companies incorporate ethical considerations into their decision-making processes.

## Data Availability

The datasets presented in this article are not readily available because De-identified interview data are available from the corresponding author on reasonable request. Access is subject to a data sharing agreement and ethics committee approval, and is granted only where sharing would not compromise participant confidentiality. Full raw transcripts cannot be shared, as they contain identifying information protected under the consent terms. Requests to access the datasets should be directed to Ece Deveci, dr.ecedeveci@gmail.com.

## References

[1] Buruk B Yildiz A Gürcan G Özüçetin B Şekerlisoy MB Yoldaş S . Case Discussions. In: Ekmekci, P.E. (eds) Ethical Dilemma in Psychiatry. Springer, Cham. (2024). doi: 10.1007/978-3-031-56211-2_6

[2] Beauchamp TL Childress JF . Principles of Biomedical Ethics. New York, Oxford University Press (2013).

[3] Radden J . Psychiatric ethics. Bioethics. (2002) 16:397–411. doi: 10.1111/1467-8519.00298 12472087

[4] Ekmekci PE Gürcan G Atalay B Bilgili F Kavas MV . Psikiyatride zorla yatış/zorla tedavi ve etik yaklaşımlar: Geleneksel derleme. Turkiye Klinikleri Tip Etigi-Hukuku-Tarihi Dergisi. (2025) 33:164–74.

[5] Murphy K Di Ruggiero E Upshur R Willison DJ Malhotra N Cai JC . Artificial intelligence for good health: A scoping review of the ethics literature. BMC Med Ethics. (2021) 22. doi: 10.1186/s12910-021-00627-6 33588803 PMC7885243

[6] Lee EE Torous J De Choudhury M Depp CA Graham SA Kim HC . Artificial intelligence for mental health care: Clinical applications, barriers, and facilitators. Biol Psychiatry Cognit Neurosci Neuroimaging. (2021) 6:856–64. doi: 10.1016/j.bpsc.2021.02.001 33571718 PMC8349367

[7] Hariman K Ventriglio A Bhugra D . The future of digital psychiatry. Curr Psychiatry Rep. (2019) 21. doi: 10.1007/s11920-019-1074-4 31410728

[8] Espejo G Reiner W Wenzinger M . Exploring the role of artificial intelligence in mental healthcare: Progress, pitfalls, and promises. Cureus. (2023) 15:e44748. doi: 10.7759/cureus.44748 37809254 PMC10556257

[9] Buruk B Ekmekci PE Arda B . A critical perspective on guidelines for responsible and trustworthy artificial intelligence. Med Health Care Philos. (2020) 23:387–99. doi: 10.1007/s11019-020-09948-1 32236794

[10] BBC News . Mothers say AI chatbots encouraged their sons to kill themselves (2025). Available online at: https://www.bbc.com/news/articles/ce3xgwyywe4o (Accessed February 20, 2026).

[11] Morley J Machado CCV Burr C Cowls J Joshi I Taddeo M . The ethics of AI in health care: A mapping review. SSRN Electron J. (2020). doi: 10.2139/ssrn.3360560 32702587

[12] Deveci E Ekmekci PE . Ethical evaluation of artificial intelligence supported psychiatric applications [Oral presentation].29th World Congress on Medical Law, World Association for Medical Law, Istanbul, Türkiye (2020).

[13] Zafar F Fakhare Alam L Vivas RR Wang J Whei SJ Mehmood S . The role of AI in identifying depression and anxiety: A comprehensive review. Cureus. (2024) 16. doi: 10.7759/cureus.56472 38638735 PMC11025697

[14] Fiske A Henningsen P Buyx A . Your robot therapist will see you now: Ethical implications of embodied AI in psychiatry, psychology, and psychotherapy. J Med Internet Res. (2019) 21:e13216. doi: 10.2196/13216 31094356 PMC6532335

[15] Stix C . 3 ways AI could help our mental health. World Economic Forum (2018). Available online at: https://www.weforum.org (Accessed December 15, 2025).

[16] Croes EAJ Antheunis ML van der Lee C de Wit JMS . Digital confessions: The willingness to disclose intimate information to a chatbot and its impact on emotional well-being. Interact Comput. (2024) 36:279. doi: 10.1093/iwc/iwae016

[17] Feng Y Hang Y Wu W Song X Xiao X Dong F . Effectiveness of AI-driven conversational agents in improving mental health among young people: Systematic review and meta-analysis. J Med Internet Res. (2025) 27:e69639. doi: 10.2196/69639 40367506 PMC12120367

[18] Cruz-Gonzalez P He AWJ Lam EPP Ng IMC Li MW Hou R . Artificial intelligence in mental health care: A systematic review of diagnosis, monitoring, and intervention applications. Psychol Med. (2025) 55:e18. doi: 10.1017/S0033291724003295 39911020 PMC12017374

[19] Stade EC Stirman SW Ungar LH Boland CL Schwartz HA Yaden DB . Large language models could change the future of behavioral healthcare: A proposal for responsible development and evaluation. NPJ Ment Health Res. (2024) 3:12. doi: 10.1038/s44184-024-00056-z 38609507 PMC10987499

[20] Alfano L Malcotti I Ciliberti R . Psychotherapy, artificial intelligence and adolescents: Ethical aspects. J Prev Med Hyg. (2024) 64:E438–42. 10.15167/2421-4248/jpmh2023.64.4.3135 PMC10876024 38379752

[21] Sedlakova J Trachsel M . Conversational AI in psychotherapy: A new therapeutic tool or agent? Am J Bioeth. (2023) 23:4–13. doi: 10.1080/15265161.2022.2048739 35362368

[22] Char DS Abramoff MD Feudtner C . Identifying ethical considerations for machine learning healthcare applications. Am J Bioeth. (2020) 20:7–17. doi: 10.1080/15265161.2020.1819469 33103967 PMC7737650

[23] Stokel-Walker C . AI-driven psychosis and suicide are on the rise, but what happens if we turn the chatbots off? BMJ. (2025) 391:r2239. doi: 10.1136/bmj.r223 41136240

[24] Ekmekci PE Crawley FP Gültekin E Arpaci S Nagabhatla N Yimam SM . A white paper on the ethics and governance of sentient AI: Navigating advanced technologies in health data spaces and systems. Zenodo. (2025). doi: 10.5281/zenodo.16688705 42270437

[25] Villongco C Khan F . Sorry I didn’t hear you”: The ethics of voice computing and AI in high-risk mental health populations. AJOB Neurosci. (2020) 11:105–12. 10.1080/21507740.2020.1740355 32228383

[26] Nazer LH Zatarah R Waldrip S Ke JXC Moukheiber M Khanna AK . Bias in artificial intelligence algorithms and recommendations for mitigation. PloS Digit Health. (2023) 2:e0000278. doi: 10.1371/journal.pdig.0000278 37347721 PMC10287014

[27] Ekmekci PE Arda B . Artificial intelligence and bioethics. In SpringerBriefs in ethics (2020). doi: 10.1007/978-3-030-52448-7

[28] Cresswell K Cunningham-Burley S Sheikh A . Health care robotics: Qualitative exploration of key challenges and future directions. J Med Internet Res. (2018) 20:e10410. doi: 10.2196/10410 29973336 PMC6053611

[29] Malouin-Lachance A Capolupo J Laplante C Hudon A . Does the digital therapeutic alliance exist? JMIR Ment Health. (2025) 12:e69294. doi: 10.2196/69294 39924298 PMC11830484

[30] Mondragón-Barrios L Bustamante Cabrera GI Marti M Muñoz Del Carpio Toia A . Ethical challenges of using artificial intelligence in suicide prevention: A literature review. New Bioeth. (2026), 1–19. doi: 10.1080/20502877.2026.2620302 41755402

[31] Abu-Mahfouz MS AlFehaid S Burqan HM El Arab RA . Artificial intelligence in mental health care: A scoping review of reviews. Front Psychiatry. (2026) 17:1688043. doi: 10.3389/fpsyt.2026.1688043 41853071 PMC12993279

[32] Kolding S Lundin RM Hansen L Østergaard SD . Use of generative artificial intelligence (AI) in psychiatry and mental health care: A systematic review. Acta Neuropsychiatr. (2025) 37:e37. doi: 10.1017/neu.2024.50 39523628 PMC13130260

[33] Monteith S Glenn T Geddes JR Whybrow PC Achtyes E Bauer M . Artificial intelligence and increasing misinformation. Br J Psychiatry. (2024) 224:33–5. Available online at: https://doi:10.1192/bjp.2023.136 (Accessed January 3, 2026). 10.1192/bjp.2023.136 37881016

[34] Baydili İ Taşcı B Taşcı G . Artificial intelligence in psychiatry: A review of biological and behavioral data analyses. Diagnostics. (2025) 15:434. doi: 10.3390/diagnostics15040434 40002587 PMC11854694

[35] Boucher EM Harake NR Ward HE Stoeckl SE Vargas J Minkel J . Artificially intelligent chatbots in digital mental health interventions: A review. Expert Rev Med Devices. (2021) 18:37–49. doi: 10.1080/17434440.2021.2013200 34872429

[36] Dehbozorgi R Zangeneh S Khooshab E Hafezi Nia D Hanif HR Samian P . The application of artificial intelligence in the field of mental health: A systematic review. BMC Psychiatry. (2025) 25:132. doi: 10.1186/s12888-025-06483-2 39953464 PMC11829440

[37] World Health Organization . Ethics and Governance of Artificial Intelligence for Health: WHO Guidance (2021). Available online at: https://iris.who.int/handle/10665/350567 (Accessed December 15, 2025).

[38] United Nations Educational, Scientific and Cultural Organization . Recommendation on the Ethics of Artificial Intelligence (2021). Available online at: https://unesdoc.unesco.org/ark:/48223/pf0000381137 (Accessed December 22, 2025).

[39] Leung WKC Lam SC Chan BCL Chow JNL Wong YYY Ng F . Chatbot interventions for improving mental health among people in Asia: A systematic review and meta-analysis of randomized controlled trials. BMJ Health Care Inform. (2026) 33:e101479. doi: 10.1136/bmjhci-2025-101479 41534952 PMC12815185

